# The TB REACH Initiative: Supporting TB Elimination Efforts in the Asia-Pacific

**DOI:** 10.3390/tropicalmed5040164

**Published:** 2020-10-26

**Authors:** Jacob Creswell, Amera Khan, Mirjam I Bakker, Miranda Brouwer, Vishnu Vardhan Kamineni, Christina Mergenthaler, Marina Smelyanskaya, Zhi Zhen Qin, Oriol Ramis, Robert Stevens, K Srikanth Reddy, Lucie Blok

**Affiliations:** 1 Stop TB Partnership, 1218 Geneva, Switzerland; amerak@stoptb.org (A.K.); m.smelyanskaya@gmail.com (M.S.); zhizhenq@stoptb.org (Z.Z.Q.); 2 KIT Royal Tropical Institute, 1092 Amsterdam, The Netherlands; M.Bakker@kit.nl (M.I.B.); C.Mergenthaler@kit.nl (C.M.); L.Blok@kit.nl (L.B.); 3 PHTB Consult, 5014 DN Tilburg, The Netherlands; phtbconsult@gmail.com; 4 Independent Consultant, Bangalore 560094, India; vvkamineni@gmail.com; 5 Epirus, 08034 Barcelona, Spain; oriolramis@gmail.com; 6 Independent Consultant, Manchester HX7 6AL, UK; robert.hartley.stevens@gmail.com; 7 Global Affairs Canada, Global Health and Nutrition Bureau, Ottawa K1A 0G2, ON, Canada; SKondreddy@bruyere.org

**Keywords:** tuberculosis, active case finding, TB preventive therapy, innovation, End TB, TB REACH

## Abstract

After many years of TB ‘control’ and incremental progress, the TB community is talking about ending the disease, yet this will only be possible with a shift in the way we approach the TB response. While the Asia-Pacific region has the highest TB burden worldwide, it also has the opportunity to lead the quest to end TB by embracing the four areas laid out in this series: using data to target hotspots, initiating active case finding, provisioning preventive TB treatment, and employing a biosocial approach. The Stop TB Partnership’s TB REACH initiative provides a platform to support partners in the development, evaluation and scale-up of new and innovative technologies and approaches to advance TB programs. We present several approaches TB REACH is taking to support its partners in the Asia-Pacific and globally to advance our collective response to end TB.

## 1. Introduction

Since the tuberculosis (TB) epidemic was declared an emergency by the World Health Organization (WHO) in the 1990s, different global TB strategies, each growing more comprehensive and ambitious in their plans and goals, have been developed and deployed. Moving from a focus on adults with TB seeking care in the public sector, strategies have evolved to provide more emphasis on children, people living with HIV, engaging the private sector, involving communities, pro-actively reaching out to people with limited access to care and addressing catastrophic costs incurred by people who have TB [1,2,3]. Once aiming to identify 70% of incident TB cases, we now understand this will not suffice to end TB and new targets call for universal access to TB care and prevention [4,5,6]. In 2018, the TB community came together at a UN High Level Meeting (UNHLM) to agree on ambitious global targets and called for an end to the epidemic in the next decade [3,7,8]. Business as usual approaches will not be sufficient to reach these targets, and this is what drives Stop TB Partnership’s Global Plan: The Paradigm Shift [7].

The TB community has been criticized for lacking ambition and being timid in its approach to combatting the disease [9,10]. The current series of this journal, Innovation and Evidence for Achieving TB Elimination in the Asia–Pacific Region, brings together several important topics for the TB community. The Asia-Pacific region accounts for more than two thirds of incident TB globally (using generally accepted countries rather than WHO’s grouping) [11,12]. However, the region has also been a leader in innovation and research in the TB field. Recent large increases in case notification primarily came from this region and bold new research is highlighting the path towards ending TB [11,13,14]. Strongly correlated with reductions in TB incidence, is socio-economic development and the Asia-Pacific region is also leading the world in GDP growth [15]. Below, we present how the Stop TB Partnership’s TB REACH initiative has addressed the different areas outlined in the Lancet Series “How to Eliminate Tuberculosis” [16] with particular attention paid to the Asia-Pacific.

## 2. The TB REACH Initiative

**Figure 1:**
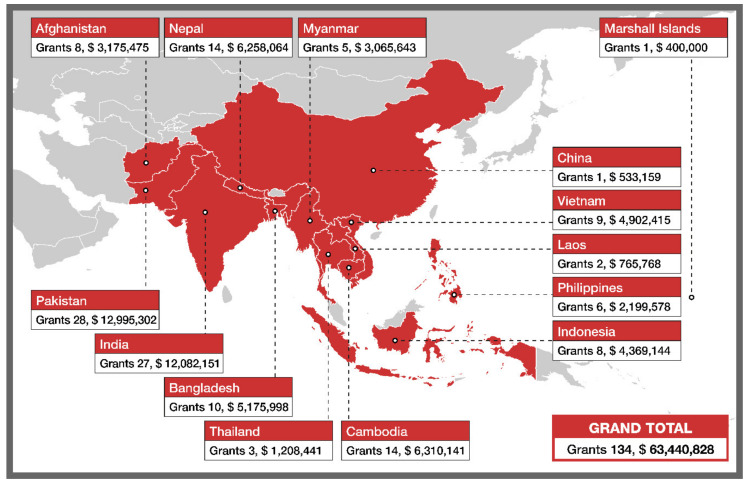
Map of TB REACH grants in the Asia-Pacific region.

The Stop TB Partnership’s TB REACH initiative [17] was established in 2009 to bring new ideas and thinking to promote bold action in the fight against TB. With foundational support from Global Affairs Canada, and additional support from USAID and The Bill and Melinda Gates Foundation, TB REACH provides rapid funding to partners to quickly implement case finding and treatment support interventions whilst conducting continuous monitoring and evaluation. Despite a focus on service delivery, each TB REACH project is given strong independent monitoring and evaluation support to track historical and prospective TB notification data as well as intervention specific indicators, so results are rigorously documented. TB REACH works with innovators and grassroots organizations, testing new approaches and technologies that many traditional donors, are less inclined to support. With eight waves of funding, TB REACH has provided USD 63.4 million to countries in the Asia-Pacific region through 134 grants (Figure 1). 

Unlike other funding mechanisms, TB REACH is not constrained by WHO guidelines as to what it can fund, which allows implementers to take risks and test new approaches or technologies. For example, TB REACH supported Xpert testing in Tanzania in mobile vans before WHO guidance was issued on the assay in 2011. The assay was used by other TB REACH projects as a front-line diagnostic while most countries were still using it only as a drug sensitivity test [18,19]. Additionally, TB REACH efforts in Nigeria and Cambodia presented interesting results from a novel pooled sputum strategy to save Xpert cartridge costs and time [20,21]. While the use of artificial intelligence (AI) in health has gained interest recently, TB REACH has been supporting AI to read chest x-rays (CXR) well before this approach was reviewed by WHO [22,23,24]. Current projects are testing new handheld X-ray machines that can be brought into communities for screening. 

TB REACH projects are limited in both time and scope. Projects generally last between 12–18 months and their funding cannot exceed USD 1 million. These limitations create the need for longer term support from governments and other donors to scale-up and sustain successful interventions. As such, TB REACH has worked with partners and donors to stimulate the adoption of new and innovative approaches by national TB programs, the Global Fund [25], and Unitaid among others [26]. 

Much of the data and examples included in this article come from experiences and results of TB REACH projects. Where possible, data has been referenced, but in some instances, results have not been published yet and been abstracted from project reports.

## 3. Data and Hotspots

The 3 million people with TB who are missed every year by routine health programs, “the missing millions”, are at the center of global discussions. At the national level, treatment coverage quantifies how well TB programs are reaching all people with TB [11]. However, these numbers fail to capture the substantial heterogeneity in treatment coverage at regional or district levels. This heterogeneity exists because of geographic features, demographics, key populations and other factors such as access to health care [27,28]. Furthermore, because people with TB are not homogenously distributed across geographical areas, mapping hotspots to help identify where best to focus active case finding (ACF) efforts is critical [29]. Often, TB REACH projects are specifically designed to focus on these hotspot areas and key populations, such as sex workers, transgender populations, people who inject drugs, prisoners, migrants, miners, ethnic minorities and indigenous populations, and other poor and/or remote communities, who have poor access to care and high burdens of TB [30]. These projects use intervention data to improve case finding as part of a rigorous monitoring and evaluation process. The continued monitoring and evaluation of TB REACH interventions ensures an understanding of the target populations demographics and specifically, how many people are reached, screened, tested and diagnosed, and where people drop out of the care cascade. A number of mobile screening applications to track people through the care cascade as well as systems to track Xpert testing have been developed [31,32].

## 4. Active Case Finding

Passive case finding (PCF) is the standard approach for TB programs globally. PCF relies on people who have chest symptoms to visit diagnostic facilities and be tested, generally with smear microscopy. While the approach is inexpensive, and can reach large parts of the population, it often misses many groups such as children, people with HIV, and many of the key populations mentioned above who have difficulties accessing care because of stigma, financial, structural, cultural and/or socio-economic barriers [5,6]. Ten years ago, TB REACH began to support innovative approaches to improve case detection including ACF programs which involve moving outside the health facility to reach people who are ill [33]. ACF often uses community members to conduct activities, and increasingly has employed CXR to identify people with TB who do not complain about symptoms. ACF is a complement to routine PCF and usually is measured by how many undiagnosed people with TB are identified and how this impacts the total notifications in a population [34]. Some differences between ACF and PCF are presented in Table 1. ACF is now an integral part of many national strategic plans for TB [34,35,36]. Here, and in this Series, we document numerous examples demonstrating the power of ACF in the Asia-Pacific region. In Indonesia, a community-based organization (CBO) increased TB notification numbers significantly through ACF initiatives aimed at remote island populations [37]. Similar increases have been attributed to ACF initiatives in Pakistan [38,39] and Cambodia [40].

Contact investigation is a core component of ACF. Although contact investigation has not lead to large increases in case notifications, it does focus on a high risk group, assists in early identification, and is the main entry point for TB preventive treatment (TPT). Furthermore, when done comprehensively, people with TB identified through contact investigation can contribute more than 10% of the total case notifications in a given population [41,42]. Despite being part of WHO and country guidelines, contact investigation is not always regularly conducted in many countries in the region. 

While a systematic review has shown that ACF alone does not impact treatment outcomes [43], the outreach associated with ACF presents better opportunities to support people with TB and ensure they successfully complete treatment, often through community health workers. In India, a CBO developed a highly successful outreach effort employing local lay workers on motorcycles to visit, screen and provide treatment support to tribal communities. The results were impressive, with a first-year pilot improving case notifications by 84% and a scale-up intervention producing similar results [44]. Moreover, pre-treatment loss to follow-up and treatment outcomes improved even with the added testing and treatment burden as the lay workers supported people with TB by visiting them consistently throughout treatment. Cambodia has been one of the earliest adopters of ACF by repurposing prevalence survey equipment to reach communities with poor access to care with both CXR and modern diagnostics such as Xpert [20,40,45,46]. While it is clear that ACF reaches people with TB earlier and can greatly improve the numbers of people treated [47,48], it is also clear that ACF alone will not be enough to end TB.

## 5. Treating TB Infection

In this series, Harries et al. presents a strong argument for the importance of including treating TB infection as we move to end TB [49]. We note that while ACF has found a strong following in the last ten years, the scale up of TPT has seen many obstacles. Harries et al. describe several challenges for TPT scale up including imperfect and expensive diagnostic tests for TB infection, long regimens, high pill burden, expensive shorter regimens, and limited recommendations on risk groups to receive treatment. However, there are a few initiatives trying to address these issues. In many cases, ACF in the form of contact investigation will be a necessary precursor to successful TPT. Numerous early adopters, such as the Zero TB Cities Initiative [50], are combining ACF and TPT to move more quickly towards the goal of ending TB. On the islands of Cu Lao Cham and Cat Ba in Viet Nam, community screening campaigns called SWEEP-TB integrated ACF and TB infection testing and treatment to create islands of elimination. These campaigns consisted of community mobilization and TB infection testing, using either the tuberculin skin test (TST) or the QuantiFERON-TB Gold Plus (QFT), in public places of congregation and subsequent door-to-door campaigns. Two days later, participants presented for evaluation of the TST or QFT results and CXR screening for active TB disease and were placed on either TB treatment or TPT as appropriate. These efforts achieved an estimated 72% population coverage with a cumulative TB infection testing of 4782 people and screening over 3100 people by CXR for the detection and treatment of 20 TB patients, two individuals with multidrug-resistant TB, and 1494 persons with TB infection. Repeat visits will attempt to document the impact of the campaigns. In the Marshall Islands, similar work was conducted combining ACF and TPT to eliminate TB from the islands. In the process, the intervention treated 4237 people with TPT and 305 for active disease [51]. Projects in other countries in the region have also shown great promise including large increases in childhood TPT enrolment in Mymensingh, Bangladesh working with both public and private providers as well as the introduction of new shorter regimens. In Indonesia, large scale Zero TB efforts are getting underway as part of a TB REACH Wave 7 project in Yogyakarta which seeks to massively scale up new shorter TPT regimens by combining ACF, and integration with community-based Maternal Child Health and Sexual Reproductive Health activities. These initiatives on TPT will identify program bottlenecks and allow TB programs to address issues during the impending scale-up of prevention efforts to meet the ambitious targets set out at the UNHLM [8]. 

## 6. Biosocial Approaches

TB is linked to poverty; its epidemiology and biology are highly dependent on social and economic factors impacting the communities where it spreads [52]. TB also aggravates poverty due to the costs of seeking care even though diagnosis and treatment itself may be free [53]. Ending TB thus means tackling the root causes of poverty, and the causes of stigma and marginalization which includes gender inequality, discrimination, racial and ethnic biases, and others. The difficulty of navigating the health care sector can add additional costs and result in people to drop out of the care cascade [54]. TB REACH supported projects in India using Accredited Social Health Activists (commonly known as ASHAs), an existing cadre working in the community as outreach workers, to guide patients through the diagnostic and treatment process, have led to substantially increased notifications [55,56]. A study in Nepal demonstrated how ACF can reduce catastrophic costs for people with TB as well as bringing health services to the community [57]. Traditional facility-based directly observed therapy requires persons with TB to regularly travel to health facilities, which can result in the incurring of travel and time costs as well as the loss of autonomy and privacy [58]. TB REACH has supported novel ways to improve treatment outcomes and enhance TB care including a portfolio of projects exploring the use of digital adherence technologies (DAT) as treatment support. The use of DAT is part of a shift towards people-centred care, empowering people with TB to take charge of their treatment and enabling health care providers to maintain contact with them and to identify individuals who may need additional support measures. In the Asia-Pacific region, current projects in Thailand, Philippines, and Bangladesh are assessing the feasibility and acceptability of these approaches as well as their effectiveness in relation to adherence and treatment outcomes with promising preliminary results [59,60,61]. In Thailand, the use of near field communication technology allowing migrants to store and share their digital treatment records, showed a 21% increase in treatment success rates in the intervention period compared to pre-intervention period.

TB programs cannot exist in a medicalized vacuum of vertical service delivery. To achieve elimination, TB programming must cut across other issues that are pertinent to communities impacted by TB. For example, malnutrition—a key symptom of poverty—can be addressed through TB programming. Interventions in Pakistan and Indonesia introduced food incentives and enablers to help people with TB and their families continue treatment and improve their overall nutritional status [37,42]. A TB REACH project in India is currently documenting the impact of India’s direct benefit transfer program plus additional food support on treatment outcomes. A CBO in Pakistan worked in collaboration with transgender women and male sex worker community leaders to provide both social and nutritional support. The project tested over 7000 people for TB and initiated anti-TB treatment for more than 600 individuals, documenting high rates of both TB and HIV among these key populations [62].

Workplaces are another setting where interventions to improve TB detection and treatment can incorporate biosocial approaches. Some settings have crowded working conditions which can increase the risk of occupational exposure to TB. Many factory workers often lack time and resources to access health care. Employees found to have TB are often at risk for discrimination, stigma, and the potential for losing their job. TB REACH has supported ACF factory-based projects for garment workers in Bangladesh and is currently supporting projects in Myanmar and in Indonesia. These projects not only focus on identifying and treating people with TB, they also work to provide education on TB awareness, stigma, and confidentiality to help empower workers to seek care in a safe environment while being able to continue to work.

During the introduction of GeneXpert technology, TB REACH made large investments in not only the diagnostic tests, but also the infrastructure, electricity, and health systems to help reach more people. While the assay is a clear improvement over smear microscopy to diagnose TB and drug resistance, its implementation in settings that lack proper power supply, space, efficient laboratory networks, and/or functional health facilities was challenging and additional investments were needed [63]. A laboratory test alone will not reach more people who are ill, and results have shown that simply placing Xpert within the health system is not enough to increase TB diagnoses [64]. New diagnostics must be placed in a functional health system including community structures/involvement with organized outreach to expand access to the technology and benefit many people [65,66]. 

TB epidemiology in the Asia Pacific Region indicates a higher risk for males [67], but women often carry the burden of TB disease differently—through being the unpaid caretakers, having their healthcare deprioritized for the benefit of their male counterparts, and carrying a heavy burden of stigma associated with the disease [68]. In addition, in many high burden TB settings women also suffer from high rates of gender-based violence, HIV and other co-occurring biosocial phenomena [69,70]. In line with the Canada’s Feminist International Assistance Policy [71] and the Sustainable Development Goals, [72] TB REACH is currently working on cultivating the links between empowering women, development and TB—an initiative first of its kind for the TB community [73]. Notably, Asia-Pacific region already has examples of strong female leadership at community level that are challenging existing gender norms and promoting gender equality through TB programming. In India, women from the community are trained to be agents of change and conduct TB education and case finding activities, as well as link people with TB to private and public health facilities for treatment [74]. In Indonesia, grassroots mobilization of female community volunteers brought a significant increase in TB notifications [37], but also strengthened the status of women in communities. In another project in Pakistan, the Kiran Sitara program trains young schoolgirls leadership and communication skills, while also advancing the TB response [75].

## 7. Conclusions

Moving from TB control to ending TB is a large shift in global policy and ambition. The Asia Pacific Region has embraced these ideas at the highest political levels [76,77]. To work towards ending TB, the TB community needs to be innovative, bold, and try things that have never been done at scale by working to bring the highest standards of care to all of those who need it. We need to make targeted investments to reach populations missed by the current approaches. ACF will be a necessary part of reaching people with TB earlier, in greater numbers, and bending the downward curve of incidence globally. However, ACF, and other innovations must be accompanied by a scale up of TPT to stem new cases from developing among people who are infected. The TB community must also embrace interventions that address the biosocial aspects of the disease, as solely medicalized approaches are insufficient to end TB. TB REACH was envisioned to test and evaluate new approaches and set them on a path to scale. We applaud the efforts to end TB in the Asia Pacific, and globally, and will continue to support new ideas to move us closer to this goal. 

## Figures and Tables

**Table 1:** Characteristics of Passive and Active Case Finding for Tuberculosis.

Passive Case Finding	Active Case Finding
Sick individuals seek care at a health facility	Health services expanded into the community
Care is provided to those who present to the facility	Targets specific groups/populations at elevated risk of TB or with limited access to services
Presumptive TB usually defined as a prolonged cough	Presumptive TB definition often more inclusive, such as any TB symptom (no duration indication) or chest X-ray abnormal
Traditionally uses smear microscopy, and increasingly molecular assays for diagnosis	Molecular assay recommended for diagnosis to reduce risk of false-positive results
Likely to identify people with TB later in their disease progression	Likely to identify people with TB earlier in their disease progression
Low health system costs, and often high patient costs	Higher health system costs, and lower patient costs
Higher TB prevalence among those screened and tested	Usually finds lower TB prevalence among those screened and tested
Often (but not always) relies on clinical staff (e.g. doctors and nurses) to carry out diagnostic evaluations	Often (but not always) relies on lay workers or community health workers to carry out diagnostic evaluations
Contributes to routine surveillance and notification data	Often measured by number needed to screen and test, and additional TB notifications
Part of the routine care provision and seeks primarily to identify people who need TB care and treatment	Focuses on reaching people with limited access to care, interrupting transmission, and decreasing TB disease incidence
